# In between Opioid Crisis and the Need to Treat Pain, where do we Stand?

**DOI:** 10.2478/jccm-2022-0029

**Published:** 2022-11-12

**Authors:** Daniela Ionescu, Simona Margarit

**Affiliations:** 1 Department of Anesthesia and Intensive Care I, Iuliu Hatieganu University of Medicine and Pharmacy, Cluj-Napoca, Romania; 2 Department of Anesthesia and Intensive Care, O Fodor Regional Institute of Gastroenterology and Hepatology, Cluj-Napoca, Romania

Acute and chronic pain are very disturbing conditions for the patient, with numerous implications on the short- and long-term outcome of patients [1]. While acute pain is experienced mostly after surgery, and in some other medical conditions as well, chronic pain may be the symptom or the result of numerous medical conditions among which cancer, muscle-skeletal or neurodegenerative diseases and surgery or persistent inflammation are, probably, the main causes [2].

This is why guidelines, drugs and medical interventions have been proposed to treat acute and chronic pain [3, 4]. While acute pain, both mild or severe is well manageable with treatment, chronic pain may be very disturbing and debilitating for the patients, especially in patients with cancer, neurodegenerative or muscle-skeletal disorders and is generally much more difficult to treat [5].

The incidence of chronic pain is generally high. It was reported that in Europe, for example, more than 20% of population suffer from moderate to severe chronic pain, with huge medical and social costs and great impact on patients’ life [5, 6]. There are big discrepancies in the incidence of chronic pain by geographic distribution and by etiology among different continents and even between countries in Europe, that may come from accuracy of diagnosis and of reporting [1, 4, 7, 8].

This is why, in the last years, considerable efforts have been done to implement guidelines for treatment of acute and chronic pain in which central concept is the multimodal pain treatment [9,10]. In the multimodal management of pain non-steroidal agents, paracetamol, nefopam and adjuvants (gabapentinoids, clonidine, etc) are included as first lines of treatment as well as other interventional techniques according to the type and intensity of pain, co-morbidities of patients and contraindications before recommending opioids [9, 10, 11, 12, 13]. These guidelines also include evidence-based recommendations of safe practice in prescribing opioids as well as gaps that still remain in opioids prescribing practice [3, 12, 13].

Despite the availability of multimodal therapy and numerous medical interventions in pain management, opioids still play a major role in acute and chronic pain treatment. In the last near 40 years since opioids were introduced for pain therapy, these drugs remain the mainstay for moderate/severe pain treatment. In this context, in the last 30 years an exponential increase in the amount of opioids used for acute and especially for chronic pain was registered around the world, especially in US and the trend remains constant in the last 6 years including in Europe [5, 14, 15, 16, 17].

This raised the question if is pain better treated or is illicit prescription of opioids increased? Statistics in the last 20-30 years show that both of these issues are valid. In the last years, it has been reported that pain is better recognized, reported and treated, especially in cancer patients and palliative care [5,15] leading to an increase of opioid prescription.

On the other hand, an over prescription and illicit consumption has been registered especially in US [5, 8, 9, 10, 11, 12, 13, 14]. This led to opioid crisis in US, a topic in urgent need for action [5, 15, 16, 17, 18] and indeed in the last years, many publications highlighted the impact of opioid crisis and the need for action [18, 19].

Taking in consideration this global picture, the next questions are if Europe is also in an opioid crisis and where is Romania in this context? The arguments for raising the first question include the increase in opioid use in Europe in the last 20 years and a relatively increased number of death due to illicit opioid use in

some European countries, while in other countries this number decreased or was stable along the last years [15]. Recent publications have also shown that in Europe situation is different between countries, with Northern part being most affected by this statistic, while in Southern part of Europe despite a similar trend in increased use of opioids, amount of opioid remains low [15, 20]. However, it is generally accepted that Europe is not facing an opioid crisis at the moment [4, 14]. The arguments for this conclusion include the fact that in Europe opioids are prescribed mainly for acute pain and only in certain cases for chronic non-cancer and cancer pain [14], that regulations are sufficient to ensure a balance between the need of opioids for pain relief and the risk of opioid use disorder [14] and additional arguments like prescribing regulations, the shift from oral to other formulations and others [4]. All these data do not mean that Europe must not pay great attention to the danger of overprescribing opioids and needs to monitor closely this phenomenon and take early action to avoid an opioid crisis similar to the one reported in USA [4, 14, 21].

Where are we in Romania? Data published so far show that in Romania the opioid consumption is low as compared with other European countries or in a classification of all countries [4, 14]. However, due to changes in regulations starting with 2007 allowing a more facile access to opioids prescription for pain treatment and on the other side imposing strict regulation to avoid unjustified prescription or illicit consumption, opioid consumption increased [4, 14, 22], still being lower than in most of European countries. In Romania opioid consumption between 2014-2016 was of 692 s-DDD (defined daily doses for statistical purposes) per 1,000,000 inhabitants/day as compared with Germany, for example, where opioids consumption was 21,346 s-DDD per 1,000,000 inhabitants/day or Belgium with 14,892 s-DDD per 1,000,000 inhabitants/day [4, 14]. The same discrepancies of up to 40 times were reported in more recent publications between our country and those countries where opioid consumption was highest [20].

Considering these data, similar to other Eastern European or American countries, the question is if Romanian patients suffer from less pain or pain is undertreated and what action needs to be taken [23]. Of course, Romanian patients do not suffer from less pain. Before a definitive answer, similar to other countries [24, 25], we need more accurate data and surveys on patients’ pain and treatment to have a more accurate picture. For example, we need to know what is the incidence of chronic pain in Romanian population, how many patients suffer from severe pain and its etiology, what is the percentage of patients having gabapentinoids included in their pain treatment or even having multimodal therapy for their pain.

This will lead to indicating the best action to be taken to have a balance between an adequate acute and chronic pain treatment and the risk of over prescription and illicit consumption of opioids.

Finally, similar to other countries, many gaps in pain treatment still remain in our country [13]. These include to my opinion a better information of both doctors and patients on the multimodal non-opioid therapies including pain treatment modules during different residency programs, nurses information on opioids use in acute and chronic pain, specific guidelines for pain treatment in different pain generating medical conditions or patients populations (hospital accreditation programs include institutional protocols for acute and chronic pain treatment) and a good adherence to these guidelines, better knowledge on the importance of rehabilitation therapy and of multidisciplinary pain management programs for an adequate chronic pain management and last, but not least, we need extensive and accurate data on the incidence of chronic pain, on medical treatment and its effectiveness and on patients main problems regarding their pain control.
